# Human Enterovirus C105, China, 2017

**DOI:** 10.3201/eid2507.180874

**Published:** 2019-07

**Authors:** Maozhong Li, Tiegang Zhang, Cheng Gong, Aihua Li, Ming Luo, Mei Dong, Fang Huang

**Affiliations:** Beijing Center for Disease Prevention and Control, Beijing, China

**Keywords:** enterovirus, enteric infections, genotype, EV-C105, viruses, China, respiratory infections

## Abstract

We report a case of enterovirus C105 infection in an 11-year-old girl with lower respiratory tract symptoms that was identified through the Respiratory Virus Surveillance System, which covers 30 sentinel hospitals in all 16 districts of Beijing, China. The presence of this virus strain in China confirmed its geographically wide distribution.

Enteroviruses are small, nonenveloped RNA viruses that cause illnesses in humans ranging from mild to severe (*1*). Fifteen species of enterovirus are known, 7 of which are known to infect humans. These species include enterovirus A–D and rhinovirus A–C (*1*,*2*). The newly emerging genotype C105 (EV-C105) represents a novel monophyletic clade of enterovirus C; this strain was identified in 2010 in the Democratic Republic of the Congo (strain 34S) (*3**,**4*). EV-C105 cases from Italy (Pavia/8376, Pavia/9095), Romania (ROM31), the United States (USA/OK/2014-19362), New Zealand (strains not available), and Burundi (BU77, BU5) have been identified and characterized, suggesting that the spread of EV-C105 could be wider than previously hypothesized (*5*). Here, we report a detected case of EV-C105 in an 11-year-old girl with lower respiratory tract symptoms in Beijing, China.

The Beijing Center for Disease Prevention and Control established the Respiratory Virus Surveillance System (RVSS) in 2014. The RVSS tracks patients with respiratory tract infections and pneumonia in 30 sentinel hospitals throughout Beijing. The RVSS is an active system, designed to alert for future outbreaks of respiratory infections (RTIs). To study enterovirus infections, we tested 24,093 clinical specimens (nasopharyngeal swab, sputum, and alveolar lavage fluid) from patients with RTIs that were reported through RVSS during June 2014–December 2017. RVSS classifies persons <14 years of age as children and those >14 years of age as adults. The ages of the reported patients ranged from 8 months to 93 years (median 33.5 years, mean 37.9 years). 

We screened all samples using real-time PCR for influenza virus, parainfluenza virus types 1–4, respiratory syncytial virus, coronaviruses (229E, NL63, HKU1, and OC43), metapneumovirus, adenovirus, bocavirus, and enteroviruses (*6*). Overall, 445 (445/7,122; 6.2%) children and 276 (276/16,971; 1.6%) adults were positive for enterovirus or other respiratory viruses.

We further genotyped enterovirus-positive samples with primers sequentially targeting the viral protein (VP) 1 region (*7**,**8*)*.* We obtained a 699-nt amplicon of EV-C105 from a nasopharyngeal swab sample collected at the time of a hospital visit (GenBank accession no. KX910099). The patient was an 11-year-old girl with no underlying disease who was brought to the outpatient clinic of the Beijing Children’s Hospital on May 23, 2016, with an 8-day history of fever (highest temperature 38.7°C), coughing, and difficulty breathing. Blood tests in the clinic showed total leukocyte count 1.41 × 10^10^ cells/L; neutrophils, 79.2%; lymphocytes, 12.1%; total platelet count, 5.2 × 10^11^/L; and hemoglobin, 130 g/L. Chest radiographs showed thickness or turbulence in the texture in both lungs, which was diagnosed as pneumonia. She received supportive treatment and received antimicrobial drugs empirically before being sent home the same day. She was not hospitalized during her illness. According to a follow-up survey, she recovered 14 days later. We detected no other respiratory pathogens in this patient.

BLAST analysis (http://blast.ncbi.nlm.nih.gov/Blast.cgi) of the amplicon (Beijing-R2759) showed that the sequences had 98% identity with the USA/OK/2014–19362 strain (GenBank accession no. KX276189.1) and 91.4% with the reference prototype genotype (EV-C105 strain 34S; accession no. JX514943). Phylogenetic analysis of the VP1 gene performed with MEGA version 6.06 software (http://www.megasoftware.net) showed that Beijing-R2759 belonged to genotype EV-C105.

To further characterize this virus strain, we amplified the genome sequence directly from the nasopharyngeal swab sample using reverse transcription PCR with overlapping primers; we sequenced each amplicon 4 times using the Sanger method. We assembled sequences using Lasergene version 5.01 (DNAStar Inc., https://www.dnastar.com). The genome of Beijing-R2759 (GenBank accession no. MH229997) was 7,316 nt, including 6,618 nt in open reading frame. The EV-C105 polyprotein sequence for this strain shares 96.6%–99.4% amino acid identity with 8 of the EV-C105 sequences in GenBank: 96.6% with accession no. KM880097 (Burundi); 96.8% with accession no. KM880096 (Burundi); 97.7% with accession no. JX393302 (Peru); 98.5% with accession no. JX514943 (Republic of the Congo); 98.6% with accession no. KM880098 (Italy); 98.8% with accession no. KM880099 (Romania); 99.3% with accession no. KX276189 (United States); and 99.4% with accession no. KM880100 (Italy). 

The full length of the Beijing-R2759 VP1 gene was 888 nt. The deduced amino acid sequence in VP1 had 94.9%–100% identity with those from Italy, Peru, Republic of the Congo, and the United States. Alignment results analysis of VP1 aa sequences showed differences between the strains isolated in this study (Met^25^, Asp^138^, Ser^207^) and EV-C105 strain 34S (Val^25^, Glu^138^, and Ala^207^). In this study, we grouped Beijing-R2759 with the strain obtained from the United States in 2014 (Figure). We observed a similar relationship in the phylogenic tree of the VP1 gene. These findings indicate that Beijing-R2759 is closely related to the EV-C105 strain reported in the United States.

**Figure:**
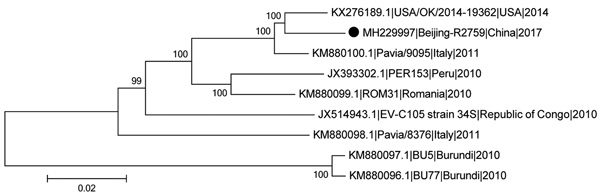
Phylogenetic tree of EV-C105 from a patient in Beijing, China (black dot) and reference isolates from different locations. We estimated the phylogenetic relationships of complete or near-complete EV-C105 genomes using the neighbor-joining method with 1,000 replicates bootstrapped by using MEGA version 6.06 software (http://www.megasoftware.net). Numbers along branches indicate bootstrap percentages. Isolates are identified by GenBank accession number, strain name, location, and year. Scale bar indicates nucleotide substitutions per site. EV-C105, enterovirus C105.

Our report confirms that the distribution of EV-C105 is geographically wider than previously believed. A greater awareness of EV-C105 may enable improved detection of this virus (*9*). In addition, our findings show the utility of the RVSS in assessing the patterns of circulation of enterovirus genotypes and detecting enterovirus outbreaks for the purpose of early warning.
